# Role of Morphology in the Diagnosis of an Unsuspected Case of Chediak-Higashi Syndrome: A Case Report

**DOI:** 10.7759/cureus.75128

**Published:** 2024-12-04

**Authors:** Rallapalli Rajyalakshmi, Pyla Kusa Raju

**Affiliations:** 1 Department of Pathology, Ranga Raya Medical College, Kakinada, IND

**Keywords:** autosomal recessive, genetics, giant granules, hemophagocytic lymphohistiocytosis, lyst gene, oculocutaneous albinism, primary immunodeficiency, vesicular trafficking

## Abstract

Chediak-Higashi syndrome (CHS) is a rare multisystem genetic disorder of childhood, caused by a defect in vesicular trafficking, which is an essential process for intracellular transport. This defect results in the formation of giant cytoplasmic granules in various cell types, including white blood cells, melanosomes, and Schwann cells. The presence of giant lysosomal granules in neutrophils and their precursors is a distinct and diagnostic feature of CHS, differentiating it from other childhood immunodeficiency disorders, such as Griscelli syndrome and Hermansky-Pudlak syndrome, which share common characteristics like albinism and increased susceptibility to fatal hemophagocytic lymphohistiocytosis. Diagnosing CHS is challenging for both clinicians and pathologists, requiring a high degree of suspicion. This case emphasizes the critical role of vigilant observation of subtle clinical and pathological features for an accurate diagnosis. We present a rare case of CHS in a six-month-old male child who presented with recurrent episodes of fever and failure to thrive. Physical examination revealed patchy gray hair on the scalp and hypopigmented patches on the lower limbs. Laboratory findings showed pancytopenia, with large, coarse cytoplasmic granules in neutrophils and their precursors on bone marrow examination - a finding diagnostic of CHS. This case highlights the importance of morphological assessment in diagnosing this rare and potentially fatal disorder, enabling early diagnosis and prompt treatment.

## Introduction

Chediak-Higashi syndrome (CHS) is a rare and fatal disorder of vesicular trafficking. The characteristic manifestations of this syndrome include primary immunodeficiency, partial oculocutaneous albinism, bleeding diathesis due to a qualitative platelet abnormality, and neuronal degeneration [1]. The fundamental defect underlying this multisystem syndrome is defective granule morphogenesis, which results in abnormal, large organelles, such as melanosomes, leukocyte granules, and platelet-dense bodies [2]. Of these, the presence of giant granules in neutrophils and myeloid precursor cells in peripheral blood and bone marrow, respectively, is diagnostic of this condition [2,3]. We encountered a rare case of CHS in a six-month-old male child, and the clinical features and hematology findings will be discussed.

## Case presentation

**Figure 1:**
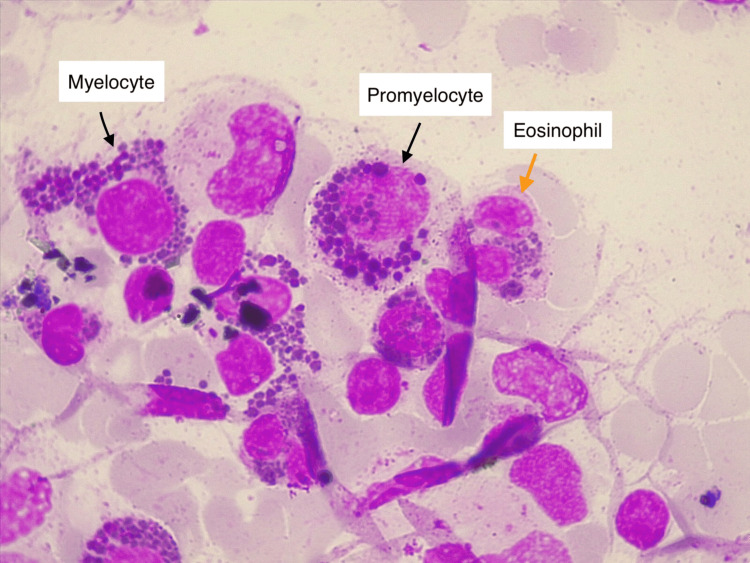
MGG-stained bone marrow aspiration smear showing characteristic giant cytoplasmic granules in a myelocyte and promyelocyte (indicated by black arrows), and in an eosinophil (indicated by an orange arrow). Few lymphocytes, as well as promyelocytes and myelocytes without granules, are also visible (1000x magnification). MGG, May-Grunwald-Giemsa

**Figure 2:**
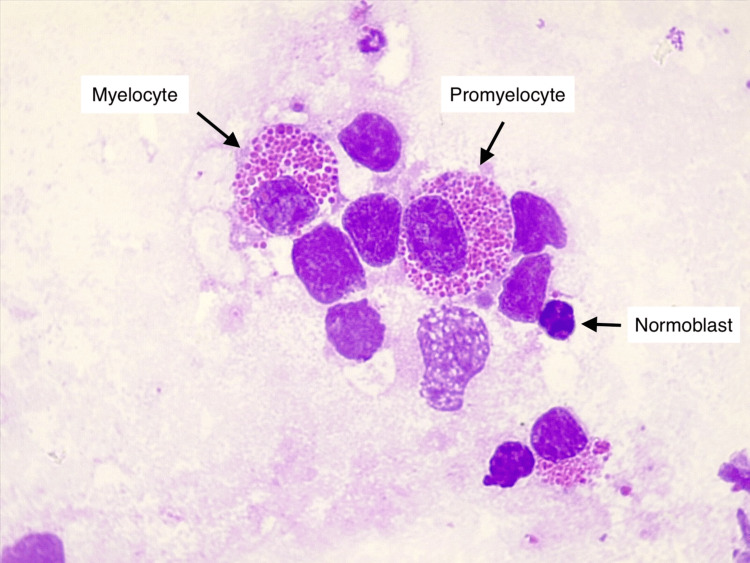
Another field of the same smear showing a promyelocyte and a myelocyte with multiple giant eosinophilic cytoplasmic granules in the cytoplasm (indicated by black arrows). Additional cells, including lymphocytes and a normoblast, are visible in the background (MGG-stained smear, 1000x magnification). MGG, May-Grunwald-Giemsa

**Figure 3:**
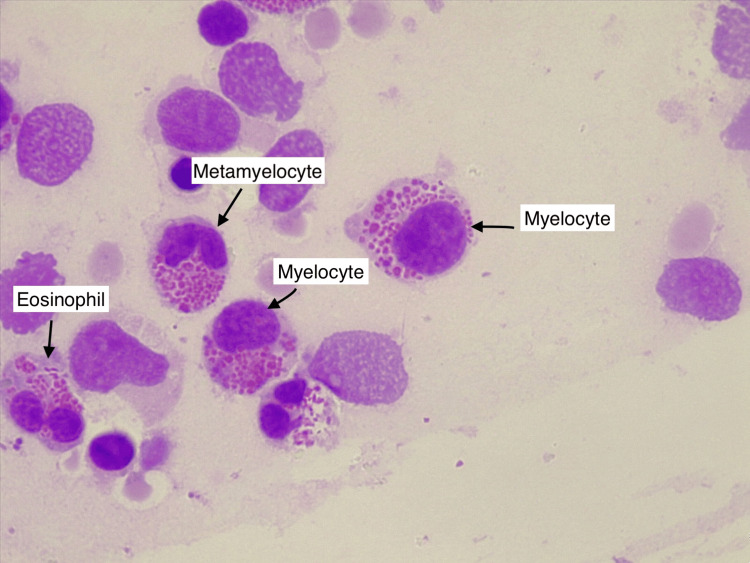
Myelocyte, metamyelocyte, and an eosinophil exhibiting giant cytoplasmic granules (indicated by black arrows) (MGG stained smear, 1000x magnification). MGG, May-Grunwald-Giemsa

A six-month-old male child, the first child of consanguineous parents, was brought to the hospital with complaints of failure to thrive and pallor for three months, along with recurrent attacks of fever for one month. The patient was thin-built and appeared paler than his parents, with hypopigmented patches on his lower limbs. He also had patchy gray hair, horizontal nystagmus, and photophobia. Ultrasound examination of the abdomen revealed hepatosplenomegaly. Blood investigations showed pancytopenia, with low hemoglobin percentage and decreased red blood cells (RBCs), white blood cells (WBCs), and platelet counts. The differential count was within normal limits. Bone marrow examination was performed from the right posterior superior iliac spine, and the May-Grunwald-Giemsa (MGG)-stained bone marrow aspiration smears were highly cellular, with myeloid, erythroid, and megakaryocyte series of cells in normal proportions. The precursor myeloid series of cells, including promyelocytes and myelocytes, and mature neutrophils and eosinophils, displayed closely packed giant granules in the cytoplasm (Figures 1-3).

Correlating these bone marrow aspiration findings with the peripheral smear and clinical features, a diagnosis of CHS was made. The patient was treated with antibiotics, nutritional supplements, and blood transfusions, and later referred to a higher institute for further management.

## Discussion

Although Beguez-César described CHS in 1943, it was Chediak and Higashi who linked this syndrome to cytoplasmic granule abnormalities in neutrophils, and it was thus named after them [2]. CHS is a rare primary immunodeficiency syndrome, with more than 500 case reports in the medical literature to date, approximately 10 of which are from India [1-5]. CHS is caused by a mutation in the *LYST*/*CHS1* gene, located on the long arm of chromosome 1 [6]. This *LYST* gene encodes the lysosomal trafficking regulator protein, which plays a crucial role in vesicular trafficking in cells across various tissues [7]. It is a large gene with several domains, such as PH, BEACH, and WD10 [1,8]. The *LYST* gene is a tumor suppressor gene and is transmitted in an autosomal recessive manner [6]. Biallelic loss of this gene results in CHS [6]. Mutational analysis of CHS has identified 74 different types of mutations, including frameshift, nonsense, missense, and splice site mutations [6-8]. Frameshift and nonsense mutations are the most common, causing a severe form of the disease in children, while missense mutations, which are less common, result in a milder form of the disease in adults [7]. 

CHS is a multisystem disease owing to the widespread effects of the mutated gene. The fundamental abnormality of unusual granule formation is induced by dysregulation of lysosomes and lysosome-related organelles, which is hypothesized to result from fusion/fission abnormalities [1,7,8]. The presence of these giant granules in various blood cells in the peripheral smear, and their precursors in the bone marrow, particularly neutrophils and myeloid precursors, is the diagnostic feature of CHS. These granules are more prominent in the bone marrow than in the peripheral blood [2]. The functional consequences of these giant granules are defective migration and impaired discharge of proteolytic enzymes, leading to impaired bactericidal activity and neutrophil chemotaxis, which makes patients susceptible to bacterial and fungal infections, particularly of the skin and respiratory tract [8,9]. Neutropenia is another consistent feature of CHS, resulting from intramedullary destruction of myeloid cells owing to the presence of large intracytoplasmic granules that make these cells less deformable. Large granules are also seen in other granulocytes, monocytes, and T and NK lymphocytes [2,7-9]. A qualitative platelet abnormality occurs due to the absence of dense bodies, resulting in poor clot formation and, consequently, prolonged bleeding time despite a normal platelet count [8]. Accumulation and abnormal clumping of melanin are seen in melanosomes due to the failure to transfer melanin to adjacent keratinocytes, leading to hypopigmented skin, hair, and eyes - a condition known as partial albinism. Hair examination shows abnormal clumping of melanin in the center, in contrast to the uniform diffuse distribution seen in normal hair. 

A fatal accelerated phase, known as hemophagocytic lymphohistiocytosis (HLH), complicates approximately 85% of CHS cases. HLH is a hyperinflammatory syndrome characterized by hypercytokinemia, also known as cytokine storm, and hemophagocytosis by activated macrophages in the liver, spleen, bone marrow, and lymph nodes. HLH is caused by impaired T lymphocyte and NK cell cytotoxicity [1,7,8,10].

CHS shares similarities with Hermansky-Pudlak syndrome, another vesicular trafficking disorder, characterized by albinism, bleeding tendency, pulmonary fibrosis, congenital neutropenia, and an increased risk of HLH. In contrast, Griscelli syndrome is a disorder of vesicular docking that causes partial albinism and immune deficiency. However, the presence of large granules in WBCs, a feature diagnostic of CHS, is not seen in the other two disorders [2,7,8,10]. Hair shaft examination shows distinct differences in pigment distribution, such as small and uniform granules in CHS, whereas large and irregular granules are seen in Griscelli syndrome. 

## Conclusions

CHS is a rare inherited disorder in children, complicated by fatal HLH. The only definitive treatment available is allogeneic bone marrow transplantation, in addition to antibiotic therapy for recurrent pyogenic infections. CHS should be suspected in children born to consanguineous parents who show albinism, nystagmus, and recurrent episodes of infections.

Although genetic testing is confirmatory, the characteristic morphology of myeloid precursor cells in the bone marrow in the present case is striking, enabling an accurate diagnosis. The present case highlights the significance of morphology for a rapid and accurate diagnosis of CHS in resource-limited settings.
